# Epigenetic disorders: Lessons from the animals–animal models in chromatinopathies

**DOI:** 10.3389/fcell.2022.979512

**Published:** 2022-09-26

**Authors:** Elisabetta Di Fede, Paolo Grazioli, Antonella Lettieri, Chiara Parodi, Silvia Castiglioni, Esi Taci, Elisa Adele Colombo, Silvia Ancona, Alberto Priori, Cristina Gervasini, Valentina Massa

**Affiliations:** ^1^ Department of Health Sciences, Università Degli Studi di Milano, Milan, Italy; ^2^ “Aldo Ravelli” Center for Neurotechnology and Experimental Brain Therapeutics, Università Degli Studi di Milano, Milan, Italy

**Keywords:** chromatinopathies, animal models, rare diseases, mus musculus, drosophila melanogaster, *Danio rerio*

## Abstract

Chromatinopathies are defined as genetic disorders caused by mutations in genes coding for protein involved in the chromatin state balance. So far 82 human conditions have been described belonging to this group of congenital disorders, sharing some molecular features and clinical signs. For almost all of these conditions, no specific treatment is available. For better understanding the molecular cascade caused by chromatin imbalance and for envisaging possible therapeutic strategies it is fundamental to combine clinical and basic research studies. To this end, animal modelling systems represent an invaluable tool to study chromatinopathies. In this review, we focused on available data in the literature of animal models mimicking the human genetic conditions. Importantly, affected organs and abnormalities are shared in the different animal models and most of these abnormalities are reported as clinical manifestation, underlying the parallelism between clinics and translational research.

## Introduction

Rare diseases are defined as conditions having a prevalence lower than 1:2000 and, nevertheless, they are estimated to be over 6,000, deeply impacting patients, families, caregivers, and health systems (Eurordis 2005). Among rare diseases, some have recently been ascribed to the so-called chromatinopathies, a heterogeneous group of Mendelian disorders affecting the epigenetic machinery (Fahrner and Bjornsson, 2014). By 2019, 70 epigenetic machinery genes have been identified, when mutated these genes are responsible for 82 human conditions. These genes were further expanded to 295 by Bjornsson and collegues (Boukas et al., 2019), and a review on monogenetic neurodevelopmental disorders (Ciptasari and van Bokhoven, 2020) is available. In this review we will focus on the 70 firstly described and better characterized causative genes. Such genes code for different epigenetic components presenting protein domains exerting writer, eraser, reader, and remodeler activities or a combination of these functions (e.g., a protein could include both a writer and a reader domain) (Fahrner and Bjornsson, 2019). Patients affected by chromatinopathies display shared clinical features, such as intellectual disability (ID) and abnormal growth, and shared etiology, which mainly relies on disruption of dosage-sensitive genes leading to haploinsufficiency (Bjornsson, 2015; Fahrner and Bjornsson, 2019).

Considering the advent of next generation sequencing (NGS) technologies, which led to the identification of a growing number of candidate variants, validation of pathogenicity can be difficult. Considering that many of the epigenetic machinery disorders are rare and/or “ultrarare”, human-based studies are often intrinsically challenging (Shen et al., 2015).

Among *in vivo* models, mice (*Mus musculus*) are the most commonly used for human genetic diseases mainly for their homology to human genome, their size, their strains that are highly inbred giving uniformed experimental conditions and reproducibility, their lifespan and for the possibility to perform genetic manipulation to obtain models of monogenic diseases, through available transgenic technologies (Dow and Lowe, 2012). Despite these advantages, mice have high husbandry costs which make them suboptimal for candidate variants assessment and drug screening. For these purposes fruit flies (*Drosophila melanogaster*) represent another valid option, considering the rapid generation time, relatively low-cost housing, and high experimental manipulability, even if evolutionary more distant from humans compared to mammals (Moulton and Letsou, 2016). Another animal commonly used for high-throughput studies is zebrafish (*Danio rerio*), which is a vertebrate with a high homology in the genome. It can be exploited for a deep characterization of disorders involving embryogenesis, due to its transparent embryos and larvae, and it represents a valid model for studying organs and structures shared with humans (Santoriello and Zon, 2012). For example, this model has been used to evaluate 3D genome organization of the epigenetic machinery (Labudina and Horsfield, 2021).

In this work we report on animal models for chromatinopathies, focusing on how these models recapitulate genotype-phenotype correlation and analyzing affected functions; moreover, we highlight the importance of an integrative approach for epigenetic machinery disorders.

## Animal models for chromatinopathies

In the last 30 years many animal models have been used to study chromatinopathies. The possibility to exploit animal models for studying the molecular mechanisms underlying a disorder, is a pivotal step for confirming etiology and pathogenic variants validation, and a valuable tool for preclinical analysis of possible therapeutic approaches. To date, the majority of translational research in this field mainly focuses on mouse (*Mus musculus*), zebrafish (*Danio rerio*) and invertebrates as fruit fly (*Drosophila melanogaster*). We also included studies on other modeling systems such as *Caenorhabditis elegans*, medaka fish (*Oryzias latipes*), *Xenopus laevis*, rat (*Rattus norvegicus*), chicken (*Gallus* gallus domesticus), rabbit (*Oryctolagus cuniculus*) and monkey (*Macaca fascicularis*). In Supplementary Table S1 animal models discussed in this review are detailed.

### Genetic heterogenous syndromes

**FIGURE 1:**
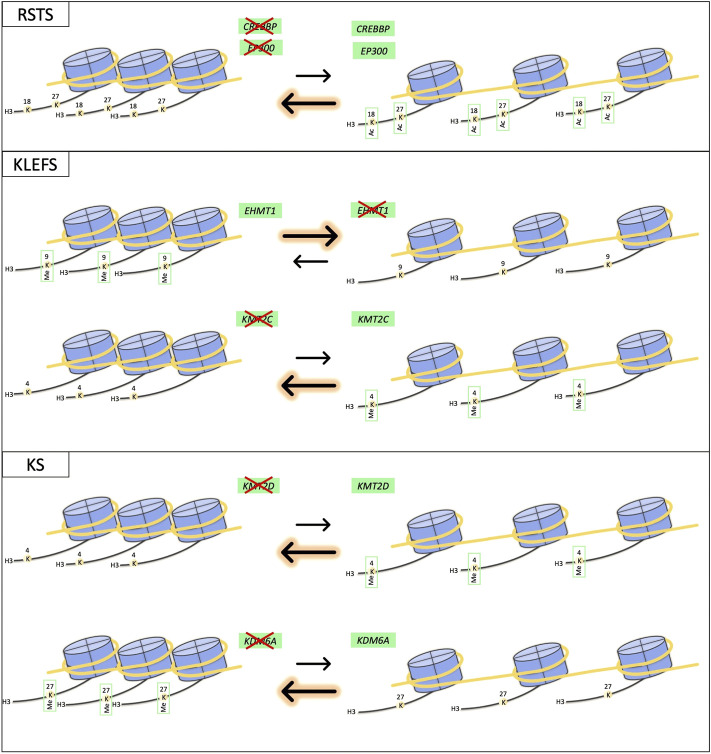
Schematic representation of effects on chromatin equilibrium for RSTS, KLEFS and KS syndromes. The drawing shows the impact of abnormal function of proteins coded by different genes having common effects on chromatin state equilibrium.

Some chromatinopathies are known to be caused by mutations in different components of the epigenetic machinery, such as Rubinstein-Taybi (RSTS1, OMIM #180849; RSTS2, OMIM #613684), Kleefstra (KLEFS1, OMIM #610253; KLEFS2, OMIM #617768) and Kabuki (KS1, OMIM #147920; KS2 OMIM #300867) syndromes. In this cases, “canonical causative genes” exert the same function on the open/close chromatin equilibrium (Figure 1). Patients affected by these syndromes show a recognizable phenotype, which can vary in severity depending on the mutated gene. Therefore, it is interesting to investigate animal models-related phenotypes recapitulating syndromes with these molecular/clinical features (below 3 examples are discussed in details) for understanding whether the human genotype-phenotype correlations are well translated *in vivo* (Supplementary Table S1).

#### Rubinstein-Taybi syndrome

RSTS is a rare neurodevelopmental multisystem malformation syndrome characterized by developmental delay and intellectual disability, growth retardation, skeletal anomalies including broad/short thumbs and/or big toes and distinctive facial features. RSTS causative genes CREBBP and EP300 code for two writers of the epigenetic machinery, CBP and p300, both histone acetyltransferases (HAT) which alterations are responsible respectively for the 60% (RSTS1) and 10% (RSTS2) of cases; p300 disruption is associated with milder phenotypes in humans (Cohen et al., 2020). Homozygous mice for Cbp or p300 (ortholog genes of human CREBBP and EP300, respectively) show embryonic lethality (Tanaka et al., 1997) and, interestingly, it has been observed also in p300 heterozygous mutants by Yao and colleagues (Yao et al., 1998). Cbp heterozygous mice are viable although displaying skeletal and cardiac abnormalities, growth retardation and memory deficits (Oike et al., 1999). In addition, craniofacial aspects and developmental delay associated with RSTS have been reported in p300 mutant mice (Viosca et al., 2010). Therefore, loss of function of Cbp and p300 leads to these similar defects in mouse models (Tanaka et al., 1997; Yao et al., 1998; Oike et al., 1999; Viosca et al., 2010) together with multilineage defects in hematopoiesis (Kung et al., 2000; Kasper et al., 2002), which in Cbp ± mice can increase the incidence of hematologic malignancies, as observed in RSTS patients (Kung et al., 2000; Boot et al., 2018). Mice lacking normal Cbp functions have been well characterized for memory and behavior capabilities, displaying synaptic plasticity deficits (Bourtchouladze et al., 2003; Alarcón et al., 2004; Wood et al., 2005), long-term memory (Tanaka et al., 1997; Oike et al., 1999; Korzus et al., 2004; Wood et al., 2006) and neuroadaptation impairment (Lopez-Atalaya et al., 2011), ASD-relevant repetitive behaviors, hyperactivity, and social interaction deficits (Zheng et al., 2016). Furthermore, Cbp seems to play a role in energy homeostasis, with mice showing increased insulin sensitivity and glucose tolerance (Yamauchi et al., 2002). In zebrafish, specific inhibition of cbp/p300 leads to a muscular dystrophy-like phenotype (Fauquier et al., 2018), while ep300 knockdown causes skeletal, cardiac and neural abnormalities (Babu et al., 2018) modelling defects present in patients. Conversely, a *Drosophila* model for EP300-related RSTS phenotype does not exist, leaving the study of dCBP mutant flies, named nejire (nejP/+), as the only option for *Drosophila* studies of RSTS. Hemizygous nej are embryonic lethal (Akimaru et al., 1997; Di Fede et al., 2021), while nejire mutants affect the eye specification and cell fate determination (Kumar et al., 2004). Similarly to what happens in mouse, knockdown of dCBP causes behavioral alterations (Boyles et al., 2010; Sethi et al., 2019), affects nervous system development (Kirilly et al., 2011) and learning, due to altered development of mushroom bodies, associative center in invertebrate brains (Li et al., 2018).

#### Kleefstra syndrome

Another chromatinopathy caused by variants in two known causative genes coding for proteins members of the epigenetic machinery is Kleefstra syndrome (KLEFS) characterized by a variable phenotype including severe intellectual disability, hypotonia, brachy (micro)cephaly, seizures, heart defects, and typical facies. Patients affected from this disorder present pathogenic variants in EHMT1 or KMT2C/MLL3, coding for two methyltransferases and epigenetic writers (respectively EHMT1 and KMT2C/MLL3), which give rise to clinically overlapping phenotypes in human (KLEFS1 and KLEFS2) (Koemans et al., 2017). However, this does not seem to be reflected in mouse models, observing on the one hand severe growth retardation and embryonic lethality in Ehmt1 knockout mice (Tachibana et al., 2005), on the other hand only partial embryonic lethality and features such as stunted growth, lower fertility, very little white fat, unusual hyperproliferation, hydronephrosis, kidney abnormalities and even ureter epithelial tumors, upon Mll3 inactivation in mice (Lee et al., 2009). Despite a mouse model heterozygous for Kmt2c (Kmt2c+/-) has never been described, Ehmt1 ± mice recapitulate closely KLEFS phenotype, displaying autistic-like features (Balemans et al., 2010), learning deficits and synaptic dysfunction (Balemans et al., 2013), delayed postnatal development and increased expression of bone developmental genes (Balemans et al., 2014), increased adult cell proliferation in the hippocampus and enhanced pattern separation ability (Benevento et al., 2017), impaired cognitive abilities and hypoactive behavior (Iacono et al., 2018). Loss of *Drosophila* EHMT1 and KMT2C orthologs, G9a and trr respectively, appears to be rather convergent in flies, leading to neurodevelopmental impairment, with defects of peripheral dendrite development, larval locomotor behavior, non-associative learning, and courtship memory observed in Ehmt1 mutants (Kramer et al., 2011), short-term memory impairment caused by trr knockdown in mushroom bodies (Koemans et al., 2017) and developmental phenotypes in trr catalytic mutant alleles after environmental stress (Rickels et al., 2017).

#### Kabuki syndrome

Kabuki syndrome (KS) is a genetic condition characterized by growth deficiency, intellectual disability, minor skeletal anomalies and distinctive facial features caused by mutations in KMT2D (60% of cases) or in KDM6A (6% of cases), coding for two different components of the epigenetic machinery, a writer and an eraser respectively. Although the affected proteins are a histone methyltransferase and a histone demethylase, they lead to indistinguishable conditions (KS1 and KS2). This could be explained by the role of these two proteins: KMT2D methylates lysine 4 of histone 3 (H3K4), while KDM6A demethylates lysine 27 of histone 3 (H3K27), both enzymes operating two epigenetic modifications associated to a common downstream effect on chromatin state (open) (Fahrner and Bjornsson, 2014). Between KS1 and KS2 only slight phenotypic differences were found in a large cohort of patients, with a major incidence of hypotonia for KMT2D patients and postnatal growth retardation for KDM6A mutation group (Miyake et al., 2013). Also, mice models for KS1 and KS2 show similarities recapitulating some aspects of the human disorder. Homozygous mice deficient for the orthologs of human KMT2D and KDM6A, Kmt2d/Mll2 and Utx respectively, show both embryonic lethality and developmental retardation (Glaser et al., 2006; Lee et al., 2012, 2013; Welstead et al., 2012; Thieme et al., 2013). In addition to these features, loss of Utx leads also to heart malformations and defective cardiovascular development (Lee et al., 2012; Shpargel et al., 2012; Welstead et al., 2012; Thieme et al., 2013), neural tube closure defects (Welstead et al., 2012) and cranioschisis (Thieme et al., 2013). Conditional mouse knockouts show that both Kmt2d and Utx are involved in myogenesis (Lee et al., 2013; Faralli et al., 2016) and in neural development since their loss can lead to hippocampal memory defects (Bjornsson, 2015) and post-migratory embryonic neural crest deficiencies (Shpargel et al., 2017). Furthermore, Utx depletion in adult females leads to myelodysplasia (Thieme et al., 2013) and in females neural crest induces severe craniofacial abnormalities (Shpargel et al., 2017), annexing tumorigenesis and dysmorphisms to the features shared with human phenotypes. Particularly, one of the most interesting mouse models is the N-ethyl-N-nitrosourea (ENU)-induced mutant mouse named bate palmas (bapa) which presents a missense mutation in Kmt2d and features such as psychomotor and behavior impairments (i.e., hypotonia), fine motor coordination and hyperactivity, closely modelling brain-associated aspects of KS1 (Yamamoto et al., 2019). To date, only *Drosophila* model for KDM6A (dUTX) loss has been studied, which results in semi-lethality and Trithorax-like phenotype (Herz et al., 2010), while both kmt2d and kdm6a zebrafish morphants have been generated, displaying skeletal and craniofacial abnormalities (Bögershausen et al., 2015; Van Laarhoven et al., 2015; Tsai et al., 2018), impairment of cardiac and angiogenic development (Van Laarhoven et al., 2015; De Los Angeles Serrano et al., 2019) and neurodevelopmental defects (Van Laarhoven et al., 2015; Tsai et al., 2018), resembling the ones observed in KS patients.

The three chromatinopathies cited above clearly represent a good example of how alterations in different genes can result in the same phenotype and, by extension, in the same disorder. This is possible in case of either common protein functions, as for RSTS and KLEFS, or different epigenetic actors, as for KS, with shared effect on chromatin balance.

### Genetic convergence for divergent phenotypes

**FIGURE 2:**
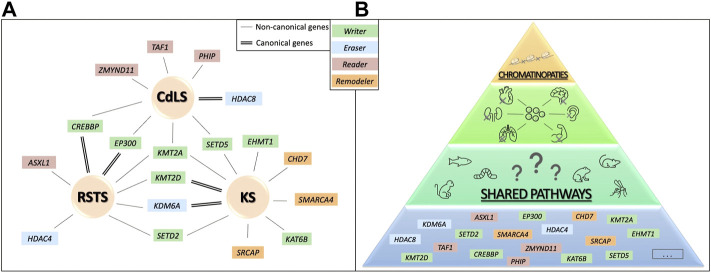
Genetic convergence for different human phenotype. In **(A)**, canonical and non-canonical causative genes are shown for RSTS, CdLS and KS. In **(B)** a schematic representation of the hypothesis of possible shared molecular pathways upon chromatin balance disruption resulting in organ abnormal development that could be studied combining animal models and human data.

Another important feature of chromatinopathies is the blurred limit among syndromes: in some cases molecular diagnosis does not coincide with the initial clinical one, complicating genotype-phenotype correlation for these disorders. Below three examples of overlapping molecular/clinical syndromes (i.e. genes coding for proteins of the epigenetic machinery “canonical” for one chromatinopathy, found altered in other chromatinopathies) are discussed in details (Figure 2A). For these cases, animal models could be an extremely relevant tool for elucidating pathogenetic mechanisms of these human disorders. &hybull;

#### Rubinstein-Taybi syndrome

Indeed, clinically diagnosed RSTS patients found negative for CREBPP/EP300 mutations resulted in presenting pathogenic variants in genes causative of other chromatinopathies such as Bohring-Opitz syndrome (BOPS, OMIM #605039) (pts #80 and #173 in (Negri et al., 2019)), KS (pt #95 in (Negri et al., 2019)), Wiedemann-Steiner syndrome (WDSTS, OMIM #605130) (pt #103 in (Negri et al., 2019) and six patients in (Di Fede et al., 2020)), Brachydactyly-Mental Retardation syndrome (BDMR, OMIM #600430) (pt #GDB1427 in (Squeo et al., 2020)) and Luscan-Lumish syndrome (LLS, OMIM #616831) (pt #18–2,798 in (Squeo et al., 2020)). Causative genes of these syndromes are not all writers of the epigenetic machinery as those responsible for RSTS (CREBBP and EP300). Indeed, only KS (KMT2D), WDSTS (KMT2A) and LLS (SETD2) genes share their epigenetic function with CREBBP and EP300, while the other KS causative gene KDM6A, BDMR gene HDAC4 and BOPS gene ASXL1 are two erasers and a reader, respectively. Despite molecular differences some patients were found to have common clinical signs and interestingly similarities can be observed in animals modelling these human disorders (Supplementary Table S1, Table 1). *In vivo* systems found in literature for all these syndromes show that if mutated, those genes impact on viability and growth (Sinclair et al., 1992; Yu et al., 1995; Akimaru et al., 1997; Tanaka et al., 1997; Sinclair et al., 1998; Yao et al., 1998; Katsani et al., 2001; Kumar et al., 2004; Vega et al., 2004; Glaser et al., 2006; Herz et al., 2010; Hu et al., 2010; Viosca et al., 2010; Lee et al., 2012; Abdel-Wahab et al., 2013; Thieme et al., 2013; Wang et al., 2014; Hsu et al., 2017; Tsai et al., 2018; Gjini et al., 2019; Liu et al., 2020), neural and brain development (Tanaka et al., 1997; Sinclair et al., 1998; Yao et al., 1998; Oike et al., 1999; Bourtchouladze et al., 2003; Wood et al., 2006; Boyles et al., 2010; Gupta et al., 2010; Hu et al., 2010; Kirilly et al., 2011; Lopez-Atalaya et al., 2011; Welstead et al., 2012; Huang et al., 2015; Van Laarhoven et al., 2015; Zheng et al., 2016; Babu et al., 2018; Li et al., 2018; Tsai et al., 2018; Yamamoto et al., 2019), hematopoiesis (Kung et al., 2000; Kasper et al., 2002; Fisher et al., 2010; Wan et al., 2011; Thieme et al., 2013; Gjini et al., 2019), and lead to skeletal and craniofacial abnormalities (Sinclair et al., 1992; Yu et al., 1995; Tanaka et al., 1997; Sinclair et al., 1998; Vega et al., 2004; Hu et al., 2010; Viosca et al., 2010; Welstead et al., 2012; Abdel-Wahab et al., 2013; Bögershausen et al., 2015; Van Laarhoven et al., 2015; Babu et al., 2018; Tsai et al., 2018), resembling human phenotypes.

**TABLE 1:** Correlation between function/organ involvement and genes belonging to the writers-erasers-readers-remodelers groups.

	Function/Organ involvement	Writers	Erasers	Readers	Remodelers
A	Viability	ASH1L - CREBBP - DNMT1 - DNMT3A- DNMT3B- EHMT1 - EP300 - EZH2 - KAT6A- KMT2A- KMT2B- KMT2D - KMT5B- PRDM16 - SETD2 - SETD5	KDM5B- KDM5C - KDM6A	ASXL1 - ASXL2 - BPTF - BRPF1 - BRWD3 - CBX2 - EED - LBR - MBD5 - MECP2 - ORC1 - PHF6 - RAI1 - RERE - SMN1 - TAF1	ATRX - CHD2 - CHD3 - CHD7 - CHD8 - SMARCA4 - SRCAP
	Neural and Brain Development	CREBBP - DNMT3A- DNMT3B- EHMT1 - EP300 - EZH2 - KAT6B- KMT2A- KMT2C - KMT2D - KMT5B- PRDM12 - SETD2 - SETD5 - WHSC1	KDM5B- KDM5C - KDM6A- PHF8	ALG13 - ASXL1 - BRPF1 - BRWD3 - EED - MBD5 - MECP2 - ORC1 - PHF6 - RAG2 - RAI1 - RERE - SMN1 - TAF1	ATRX - CHD2 - CHD7 - CHD8 - SMARCA2 - SMARCA4 - SRCAP
	Growth	ASH1L - CREBBP - DNMT1 - DNM3A- DNM3B- EHMT1 - EP300 - EZH2 - KMT2A- KMT2C - KMT2D - KMT2E - KMT5B- NSD1 - SETD2 - SETD5 - WHSC1	HDAC8 - HR - KDM5C - KDM6A	AIRE - ASXL1 - ASXL2 - BPTF - CBX2 - EED - LBR - MBD5 - MECP2 - ORC1 - RAI1 - RERE - TAF1	ATRX - CHD2
	Skeletal and Craniofacial Development	ASH1L - CREBBP - DNMT3B- EP300 - EZH2 - KAT6A- KAT6B- KMT2A- KMT2D - KMT5B- PRDM16 - WHSC1	KDM6A- HDAC4 - HDAC8 - KDM5B	ASXL1 - ASXL2 - BPTF - BRPF1 - CBX2 - EED - LBR - ORC1 - RAI1 - RERE - ZMYND11	ATRX - CHD2 - CHD7 - CHD8
	Heart and Vascular Development	CREBBP - DNMT3B- EP300 - EZH2 - KAT6A- KMT2B- KMT2D - PRDM16 - SETD2 - SETD5 - WHSC1	KDM6A	ASXL2 - BRPF1 - EED - RERE	CHD2 - CHD7 - SMARCA4
	Hematopoiesis	CREBBP - DNMT3A- EP300 - EZH2- KAT6A- KMT2A- KMT2E	KDM6A	ASXL1 - BPTF - BRPF1 - CBX2 - EED - LBR - PHF6 - TAF1	SRCAP
	Eyes	CREBBP - DNMT3A- EZH2 - KMT2B- KMT2C - KMT5B- NSD1 - SETD5 - WHSC1	KDM5B	BRWD3 - RERE - TAF1 - TDRD7	CHD7 - SMARCA4
	Memory	CREBBP - DNMT1 - DNMT3A- EHMT1 - KMT2A- KMT2B- KMT2C - KMT2D - NSD1	PHF8	BRPF1 - RAI1	CHD1 - CHD2 - CHD7 - SMARCA2
	Behavior	CREBBP - EHMT1 - KMT2C - KMT2D - SETD5	KDM5C	MECP2 - PHF6 - RAG2 - RAI1	ATRX - CHD2 - CHD8 - SMARCA2
B	Fertility	ASH1L - DNMT3A- KMT2C - KMT2E	-	AIRE - CBX2 - MORC2 - ORC1 - RAI1 - TAF1 - TDRD7	CHD1 - SRCAP

	Tumorigenesis	CREBBP - DNMT1 - DNMT3B- KMT2C	KDM6A	ASXL1 - ASXL2 - BPTF - MORC2 - MSH6 - PHF6	SMARCA4	

	Immunity	KMT2A	KDM1A	AIRE - MECP2 - RAG2 - SMN1	SMARCA4	

	Muscle	CREBBP - EP300 - KMT5B	KDM6A	SMN1	-	

	Energy Homeostasis	CREBBP - EHMT1 - SETD2	-	ASXL2	CHD8	

	Insulinemia and Glucose Homeostasis	EZH2 - KMT2B	-	ASXL2 - EED - MBD5	-	

	Gut	EZH2	-	ASXL1	CHD8	

	Obesity	EHMT1	-	RAI1 - MECP2	-	

	Kidney	NSD1	-	EED - RERE	-	

	Hair/Skin	-	HR	-	-	


#### Kabuki syndrome

As described for RSTS patients, initial clinical diagnosis for Kabuki syndrome (KS) was not always confirmed at molecular level. KS patients negative for mutations in KMT2D or KMD6A were found carriers of pathogenic variants in genes related to WDSTS, KLEFS, Mental Retardation Autosomal Dominant 23 (MRD23, OMIM #615761), Say-Barber-Biesecker-Young-Simpson or Ohdo syndrome (SBBYSS, OMIM #603736), Coffin-Siris syndrome-4 (CSS4, OMIM #614609), Floating Harbor syndrome (FLHS, OMIM #136140), CHARGE syndrome (CHARGE, OMIM #214800) and LLS (e.g. pts GDB1054, GDB1405, GDB1400, #18–2,261, GDB1128, GDB1185, GDB1311, GDB1404, GDB1422, GDB1154, GDB1406, GDB1433 in (Squeo et al., 2020)). As KMT2D, most of the causative genes of the aforementioned syndromes are writers of the epigenetic machinery (KMT2A, EHMT1, SETD5, KAT6B and SETD2), except for the remodelers SMARCA4 (CSS4), SRCAP (FLHS) and CHD7 (CHARGE). Animal models mimicking these disorders (Supplementary Table S1, Table 1) do not reach adulthood when ortholog genes are depleted, as observed in mouse (Yu et al., 1995; Bultman et al., 2000; Bosman et al., 2005; Tachibana et al., 2005; Glaser et al., 2006; Hu et al., 2010; Lee et al., 2012; Thieme et al., 2013; Osipovich et al., 2016), zebrafish (Thieme et al., 2013; Tsai et al., 2018) and fruit fly (Braun et al., 1997; Ruhf et al., 2001; Herz et al., 2010), with the only exception of KAT6B mutant mice getting through the postnatal period (Thomas et al., 2000; Merson et al., 2006). Interestingly, despite the variety of mutants used for studying these disorders, they display anomalies impacting similar neural aspects: neural tube closure in mouse (Hu et al., 2010; Welstead et al., 2012; Osipovich et al., 2016); neuronal differentiation in zebrafish (Van Laarhoven et al., 2015) and *Xenopus* (Seo et al., 2005); neural development and neurogenesis in rodents (Merson et al., 2006; Layman et al., 2009; Clayton-Smith et al., 2011; Moore et al., 2019; Sessa et al., 2019), zebrafish (Huang et al., 2015; Van Laarhoven et al., 2015; Tsai et al., 2018) and fruit flies (Braun et al., 1997; Melicharek et al., 2010; Kramer et al., 2011); cognitive ability and synaptic functions in mice (Layman et al., 2009; Gupta et al., 2010; Balemans et al., 2013; Iacono et al., 2018; Sessa et al., 2019) and *Drosophila* (Melicharek et al., 2010; Kramer et al., 2011); psychomotor functions and autistic-like behavior in mice (Balemans et al., 2010; Deliu et al., 2018; Moore et al., 2019; Sessa et al., 2019; Yamamoto et al., 2019) and *Drosophila* (Melicharek et al., 2010; Kramer et al., 2011). In addition, patients affected from syndromes such as KS, MRD32, CSS4 and CHARGE often display heart defects and cardiovascular anomalies (Kosho et al., 2014; Koemans et al., 2017; van Ravenswaaij-Arts and Martin, 2017; Powis et al., 2018) as well as animals modelling these disorders (Bosman et al., 2005; Takeuchi et al., 2011; Lee et al., 2012; Shpargel et al., 2012; Welstead et al., 2012; Thieme et al., 2013; Van Laarhoven et al., 2015; Osipovich et al., 2016; De Los Angeles Serrano et al., 2019). Although similar defects have been observed also in WDSTS, KLEFS1 and SBBYSS patients (Lemire et al., 2012; Willemsen et al., 2012; Baer et al., 2018), *in vivo* systems for these syndromes do not show anomalies in this organ.

#### Cornelia de lange syndrome

CdLS is a rare heterogenous developmental disorder characterized by multiorgan abnormalities leading to severe growth delay, distinctive facial feature, psychomotor deficit, intellectual disability, behavioral problems, and limb malformations. Patients firstly diagnosed by geneticists for having CdLS (CdLS1, OMIM #122470; CdLS2, OMIM #300590; CdLS3, OMIM #610759; CdLS4, OMIM #614701; CdLS5, OMIM #300882), not always carried mutations in one of the seven known genes causative of this syndrome. Instead, the genetic alteration can be found in a gene associated with another syndrome even though patients display typical characteristics described in the CdLS consensus (Kline et al., 2018). As a matter of fact, CdLS is now considered a “clinical spectrum” rather than an isolated syndrome with unique features (Selicorni et al., 2021). Genes found mutated in patients with an initial diagnosis of CdLS are: EP300 ((Aoi et al., 2019) - Patient 6; (Cucco et al., 2020); - Patient A; (Squeo et al., 2020); - GDB1418; (Woods et al., 2014); - Case Report)), KMT2A ((Yuan et al., 2015) - CdLS-3 (BAB4964); (Parenti et al., 2017); - Patient 12; (Aoi et al., 2019); - Patient 27; (Demir et al., 2021); - Case Report)); TAF1 ((O’Rawe et al., 2015) - Individual 4A; (Cheng et al., 2020); - Individual 13) causative of Mental Retardation X-Linked Syndromic 33 (MRXS33, OMIM #300966)); ZMYND11((Aoi et al., 2019) - Patient 53) causative of Mental Retardation Autosomal Dominant 30 (MRD30, OMIM #616083)); PHIP ((Aoi et al., 2019) - Patient 56) associated to Developmental Delay Intellectual Disability Obesity and Dysmorphism or Chung-Jansen syndrome (CHUJANS, OMIM #617991)), CREBBP ((Tang et al., 2019) - Patient 3)); SETD5 ((Squeo et al., 2020) - GDB1400) causative of MRD23). These genes, involved in chromatin regulation, are associated with syndromes different from CdLS, but studies conducted exploiting animal models highlighted some similar characteristics that can be found in the human patients affected with CdLS. Almost all animals mutated in the above mentioned genes display impaired viability or overall defects in growth (HDAC8, EP300, KMT2A, TAF1, CREBBP, SETD5) (Yu et al., 1995; Akimaru et al., 1997; Tanaka et al., 1997; Yao et al., 1998; Wassarman et al., 2000; Katsani et al., 2001; Kumar et al., 2004; Haberland et al., 2009; Viosca et al., 2010; Osipovich et al., 2016; Deliu et al., 2018; Gudmundsson et al., 2019), usually with mild to severe problems affecting the embryonic neurodevelopment (HDAC8, EP300, KMT2A, TAF1, CREBBP, SETD5) (Tanaka et al., 1997; Yao et al., 1998; Oike et al., 1999; Bourtchouladze et al., 2003; Wood et al., 2006; Haberland et al., 2009; Boyles et al., 2010; Gupta et al., 2010; Kirilly et al., 2011; Lopez-Atalaya et al., 2011; Huang et al., 2015; O’Rawe et al., 2015; Osipovich et al., 2016; Zheng et al., 2016; Babu et al., 2018; Deliu et al., 2018; Li et al., 2018; Gudmundsson et al., 2019; Sessa et al., 2019) and skeletal abnormalities often found in the craniofacial district (Yu et al., 1995; Tanaka et al., 1997; Haberland et al., 2009; Viosca et al., 2010; Babu et al., 2018; Sun et al., 2018; Sessa et al., 2019) (HDAC8, EP300, KMT2A, ZMYND11, CREBBP, SETD5) (Supplementary Table S1, Table 1).

## Lessons from the models

**FIGURE 3:**
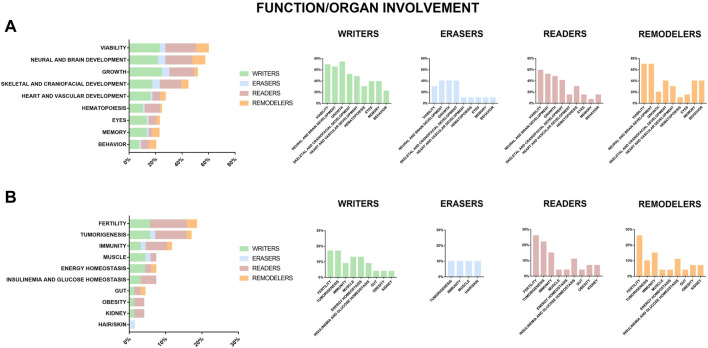
Function or organ found impaired or altered in animal models of chromatinopathies. Genes coding for writers (green), erasers (blue), readers (pink) and remodelers (orange), for which haploinsufficiency results in human abnormalities. **(A)** reports functions/organs in animal models altered in more than 20% of analyzed genes and **(B)** those in <20%.

*In vivo* systems representative of depletion or alteration of chromatin regulators, listed in Supplementary Table S1, show many parallelisms with patients affected by chromatinopathies. As shown in Figure 3 genes causative of human chromatinopathies impact common organ function/development also in different animal models. As portrayed in Figure 3A and Table 1, the most compromised functions in most animal models we found reported for all the players of the epigenetic machinery (writers, erasers, readers, and remodelers) resulted to be viability, growth, and neural and skeletal development. These features can be identified in patients who often present main clinical signs such as growth delay, intellectual disability (ID), facial dysmorphisms and skeletal anomalies (Fahrner and Bjornsson, 2014). In addition, most individuals affected by chromatinopathies have loss of function mutations leading to haploinsufficiency, due to the fundamental role of causative genes in embryonic development whose complete loss would be often at odds with life (Bjornsson, 2015). Of note, the vascular system together with organs such as heart and eyes seem particularly affected in writers models (Figure 3A), memory in writers and remodelers models (Figure 3A), while fertility resulted mostly altered in readers models (Figure 3B). Furthermore, about 20% of these *in vivo* systems display defects in hematopoiesis and altered mechanisms leading to tumorigenesis (Figure 3 and Table 1). Interestingly, although somatic mutations in genes of the epigenetic apparatus have been found in different types of tumors, cancer predisposition due to germline mutations is a feature common to some chromatinopathies. For instance, a peculiar aspect of RSTS is the increased incidence of benign and malignant tumors (Boot et al., 2018), which can be observed in the Cbp ± mouse model for this syndrome (Kung et al., 2000). Tumors susceptibility has been studied also for BOPS and was found that, although isolated reports on BOPS children seem to suggest a greater risk for Wilms tumor, sporadic malignancies in absence of other BOPS findings more frequently harbor somatic ASXL1 pathogenetic variants (Russell et al., 2018) which increase the risk of myelodysplastic syndrome as shown in mice (Abdel-Wahab et al., 2013; Wang et al., 2014). A tumor predisposition was also found in patients positive for germline mutation in MSH6 and SMARCA4 who developed respectively Hereditary nonpolyposis colorectal cancer type 5 and rhabdoid tumors (Biegel et al., 2014; Lepore Signorile et al., 2021) and this increased risk of developing malignancies was observed also in their corresponding animal models (Edelmann et al., 1997; De Wind et al., 1999; Bultman et al., 2000; Feitsma et al., 2008; Peled et al., 2010). Conversely, for Immunodeficiency-centromeric instability-facial anomalies syndrome 1 (ICF1, OMIM #242860), Borjeson-Forssmann-Lehmann syndrome (BFLS, OMIM #301900) and KS a cancer association has only been hypothesized so far, as patients who developed Hodgkin lymphoma, adrenocortical adenoma, acute lymphoblastic leukemia, Burkitt’s lymphoma and solid tumors (Ijichi et al., 1996; Scherer et al., 2003; Ehrlich et al., 2008; Chao et al., 2010a,b; Wang et al., 2019), and animal models presenting hematopoietic tumors (Shah et al., 2010; Thieme et al., 2013; Hsu et al., 2019) have been reported for these syndromes.

## Conclusion

*In vivo* models require genetic manipulation and shared homology with the human genome, leading to the possibility of mimicking the human genetic disease for studying for possibly shared molecular mechanisms responsible for clinical phenotypes and examine physically or temporarily inaccessible tissues (Figure 2B). Animal models for chromatinopathies have proved to be a valuable tool for dissecting mechanisms underlying these disorders and altered functions due to mutations in genes of the epigenetic apparatus. More variants in genes that can be grouped in the chromatinopathies are increasingly reported as well as animal models for their study. Hence, we propose a table with details of the first 70 well characterized genes, with the possibility of expanding such collection in an open science format (https://www.shorturl.at/nuV78). Importantly, affected organs and abnormalities are shared in the different animal models (listed in Supplementary Table S1), generated for a better understanding for the effects of loss or alteration of epigenetic genes (represented in Figure 3 and Table 1). Most of these abnormalities can also be found in patients affected by chromatinopathies, pointing out once again the parallelism between clinics and translational research. Importantly, for better dissecting each organ/function abnormalities in these rare conditions many studies are undergoing exploiting also stem cells and organoids for combining human data and animal model information. Interestingly, Boukas and colleagues (Boukas et al., 2019) recently demonstrated that a large subset of genes belonging to the epigenetic machinery are highly co-expressed, intolerant to variation and independently enriched for genes affecting neurological function. This suggests a link between these properties, highlighting once again the interconnection between epigenetic regulators. This aspect can be observed in applied research, where modelling disorders leads to phenotypes resembling not only the human disease but also shared features among chromatinopathies. Thus, to ensure the understanding of molecular mechanisms characterizing these disorders an integrative approach should be supported.
